# Gastrointestinal parasites in red-legged partridges (*Alectoris rufa*) hunted in Spain: a warning to game managers

**DOI:** 10.1007/s11259-022-09968-7

**Published:** 2022-07-07

**Authors:** Irene Arcenillas-Hernández, Carlos Martínez-Carrasco, Paolo Tizzani, Eduardo Berriatua, María del Rocío Ruiz de Ybáñez

**Affiliations:** 1 Department of Animal Health, Regional Campus of International Excellence ‘Campus Mare Nostrum’, University of Murcia, Murcia, Spain; 2 Department of Veterinary Science, University of Turin, 10090 Grugliasco, Italy

**Keywords:** Bioclimatic region, *Eimeria*, Helminths, Management, Parasites, Red-legged partridge

## Abstract

Red-legged partridge (*Alectoris rufa*) populations are currently declining in the Iberian Peninsula, mainly due to habitat degradation and hunting pressure. In addition, the release of farm-reared partridges may introduce pathogens, including parasites, to wild populations. The presence of digestive parasites in red-legged partridges hunted in fifteen Spanish provinces was studied. Fecal samples and gastrointestinal tracts were collected, analyzed, and the morphometric identification of parasites was carried out. *Eimeria* spp. oocysts, nematode, cestode and trematode eggs were observed in fecal samples. Adult nematodes (*Ascaridia galli*, *Ascaridia compar*, *Heterakis gallinarum*, *Heterakis tenuicauda*, *Trichostrongylus tenuis, Subulura* spp., *Cyrnea* spp. and *Aonchotheca caudinflata*), tapeworms (*Raillietina tetragona, R. echinobothrida, R. micracantha, Rhabdometra nigropunctata,* and *Choanotaenia infundibulum*), and trematodes (*Brachylaima* spp., *Brachylecithum* spp., *Dicrocoelium* spp.) were identified in the gastrointestinal tracts. Significant statistical differences were found among climatic regions in the prevalence and intensity of *Eimeria* spp. infection, median intensity and the prevalence of indirect life cycle helminths, with Southern areas always showing higher infection values. The study provides information of the health status of red-legged partridges in Spain, highlighting the risk associated with the release of farm-reared partridges for restocking purposes. This should be taken into account to improve management strategies for the long-term conservation of the species.

## Introduction

The red-legged partridge (*Alectoris rufa*) is a medium-size European galliform, with natural populations reported in Portugal, Andorra, France, Germany, Italy and Spain (Blanco-Aguiar et al. 2003; Birdlife International 2022). This species carries out an important ecological role in Mediterranean ecosystems (Díaz-Fernández et al. 2013), with a wide distribution in the Iberian Peninsula ranging from natural environments to farmland mosaics (Cabodevilla et al. 2021). In addition, the red-legged partridge is the principal prey for a large number of threatened species (Blanco-Aguiar et al. 2004; Arroyo et al. 2017). Also, the red-legged partridge has a significant socio-economic value as the main game bird species, with around 3 to 5 million of farm-reared partridges annually released in Spain for shooting (Blanco-Aguiar et al. 2003; Sánchez García-Abad et al. 2009).

During the last decade, red-legged partridge populations have declined by 40–45% in Europe (Birdlife International 2022). Agricultural intensification, with negative effects on suitable habitats for this galliform, is one of the main threats to its survival (Delibes-Mateos et al. 2012; Cabodevilla et al. 2021). On the other hand, overhunting, hybridization, predation and diseases are factors that have contributed to the reduction of red-legged partridge populations in the Iberian Peninsula (Calvete et al. 2003; Blanco-Aguiar et al. 2008; Buenestado et al. 2008, 2009; Villanúa et al. 2008; Casas and Viñuela 2010).

The main objective of hunting estate managers is to raise the availability of these birds and, therefore, populations are usually restocked with farm-reared partridges (Díaz-Fernández et al. 2013; Casas et al. 2016). Also, predator control or habitat management measures (water points, supplementary feeders or game crops) are frequently used to maintain or increase partridge population density, particularly in areas where hunting provides significant economic benefits (Gortázar et al. 2000; Arroyo et al. 2012). Specifically, water points, supplementary feeders or game crops are some of the measures used to improve the survival of released farm-reared individuals and to achieve their permanent settlement on hunting estates (Gortázar et al. 2000).

Previous studies have pointed out the differences between the parasite community of farm-reared and wild partridges (Millán et al. 2004b). Farm-reared partridges are usually infected by monoxenous nematodes such as *Ascaridia* spp., *Heterakis* spp. or *Aonchoteca caudinflata*. In case of wild individuals, heteroxenous parasites (*Cyrnea* spp., *Subulura* spp., flukes or tapeworms) are the most predominant (Millán 2009). Therefore, one of the negative effects of releasing farm-reared birds is an eventual introduction and spread of diseases to wild red-legged partridge populations. In addition, hybridization with other partridge species represent a threat for the long-term survival of this native species (Villanúa et al. 2007; Jamieson and Lacy 2012; Sánchez-Donoso et al. 2012).

Environment and climatic conditions could influence the life cycle of parasite species, and so, their ecology, survival and spread, impacting also on the host populations (Morand 2015; Brunner and Eizaguirre 2016; Holand et al. 2019). In this sense, the Iberian Peninsula has different bioclimatic zones, being Mediterranean and Atlantic areas the main bioclimatic subdivision. Mediterranean climate covers territories with warm, dry summers and cool, wet winters, while the Atlantic area is wetter and colder (Rey Benayas and Scheiner 2002). This difference in climate defines the host (Sillero et al. 2009) and parasite diversity (Sanchis-Monsonís et al. 2019).

The aim of this study was to characterize the parasites of the gastrointestinal tract of red-legged partridges from several hunting estates distributed along the Iberian Peninsula, as well as to determine the influence of some biotic (sex and age of the host) and abiotic (geographical area of provenance) characteristics on the parasite community. In addition, the relationship between the characteristics of each climatic region and the parasite richness was discussed, as well as the risks related to the release of farm-reared partridges. Our results may help to design new management recommendations to preserve natural red-legged partridge populations.

## Material & methods

### Study area and animal collection

During the winters of 2010–2013, 934 red-legged partridges shot in 139 hunting estates from 15 out of the 50 Spanish provinces were collected. The hunting estates sampled regularly restocking with farm-raised red-legged partridges, although it was not possible to obtain detailed information about the frequency of these releases. At the time of sampling, it was not possible to differentiate whether the partridges were wild or farm-reared, because the latter were not ringed. The environmental heterogeneity of the Iberian Peninsula leads to great differences between the northern area and the Mediterranean one, which occupies most of the central, eastern and southern part of the country (Olson et al. 2001). In this sense, the area of study was classified as Humid Temperate Atlantic (average annual precipitation: 1000–1600 mm; average annual temperature: 7-12ºC), Subhumid Supramediterranean (600–1000 mm; 8-13ºC), Semiarid-Mesomediterranean (350–600 mm; 13-17ºC) and Thermomediterranean (200–350 mm; 17-19ºC) (Rivas-Martínez et al. 2017).

**Fig. 1:**
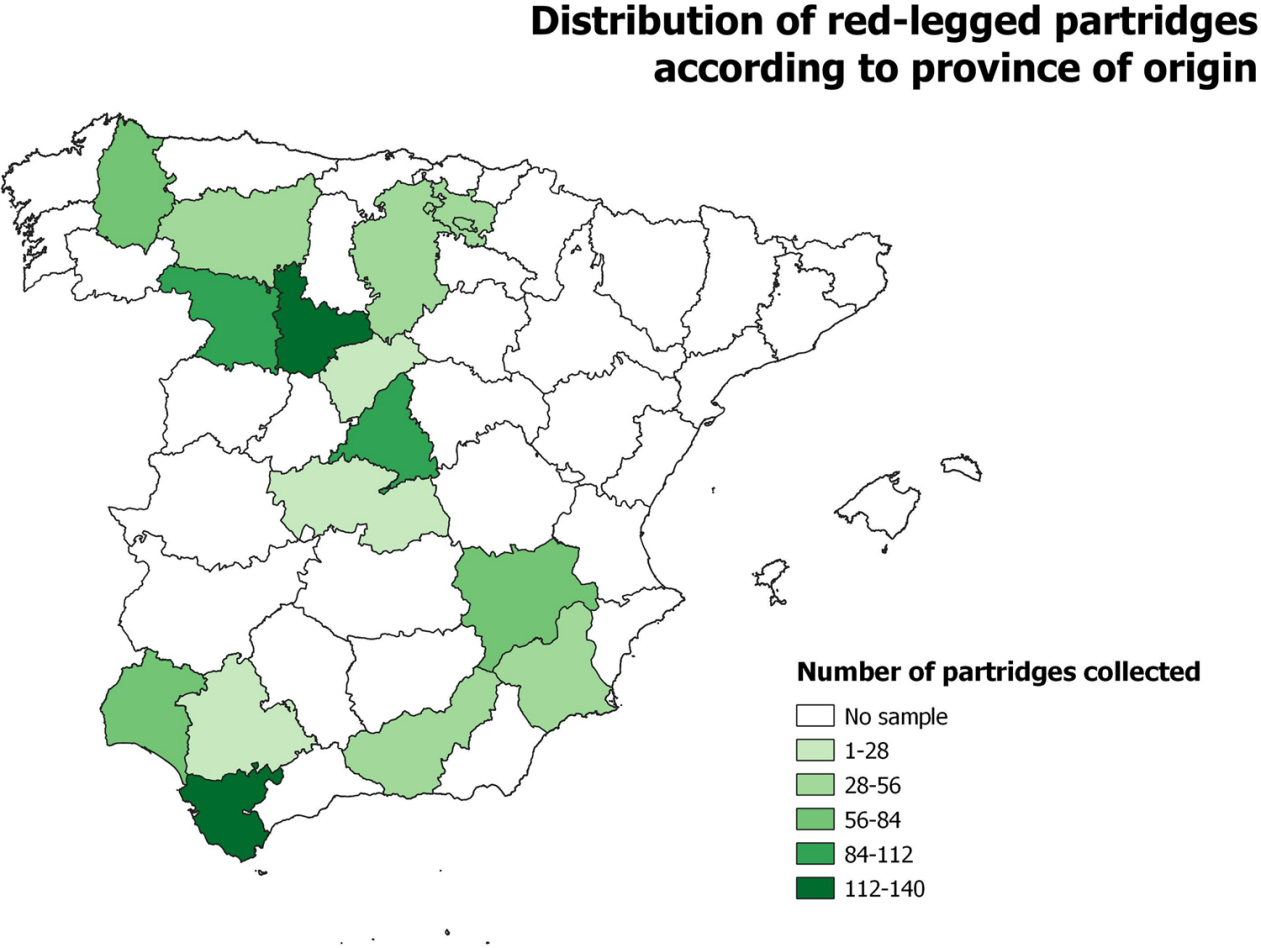
Number of red-legged partridges collected and their distribution according to the province of provenance

Red-legged partridges were categorized by sex and age following the descriptions of Sáenz de Buruaga et al. (1991). The distribution of the sample by sex, age and bioclimatic zone is shown in Table 1 and Fig. 1.

**Table 1:** Distribution of red-legged partridges (*n* = 934) attending to the sex and age of the host, as well as climate area and province of origin

Climate region^(a)^	Province	N^(c)^	Sex			Age category			
			Male	Female	NA^(b)^	Juvenile	Subadult	Adult	NA
HTA	Lugo	63	11	36	16	0	3	44	16
	Álava	40	0	0	40	0	0	0	40
SS	Segovia	6	5	1	0	1	0	5	0
	Burgos	49	1	3	45	3	0	1	45
	Valladolid	140	44	21	75	33	15	17	75
	León	53	8	5	40	3	4	6	40
SM	Toledo	25	6	4	15	4	1	5	15
	Madrid	87	11	13	63	10	2	12	63
	Zamora	91	56	35	0	22	15	54	0
	Albacete	70	27	24	19	21	0	30	19
	Granada	38	0	3	35	2	0	1	35
T	Murcia	56	26	28	2	17	32	5	2
	Huelva	81	0	0	81	0	0	0	81
	Cádiz	118	65	44	9	7	1	102	8
	Sevilla	17	0	0	17	0	0	0	17
Total		934	260	217	457	123	73	282	456

(a) *HTA* Humid Temperate Atlantic, *SS* Subhumid Supramediterranean, *SM* Semiarid-Mesomediterranean, *T* Thermomediterranean

(b) NA: data not available

(c) N: number of red-legged partridges

The gastrointestinal tract (including proventriculus, gizzard, small intestine, caeca, colon and cloaca) of 547 birds, and only the large intestine and cloaca from the remaining partridges (*n* = 387) were extracted by the hunters, individually refrigerated in plastic labelled bags and submitted to the Department of Animal Health at Murcia University, where they were frozen at -20ºC until they were analysed, which was always less than two weeks after receipt of the samples.

### Laboratory procedures

#### Coprological analysis

Of all submitted samples that contained feces in the cloaca (*n* = 927), coprological analysis was performed with both centrifugation-flotation and sedimentation techniques (MAFF 1986), using Sheather's solution (ρ = 1.27 g/ml) as flotation fluid. Two-chamber McMaster slides were filled in triplicate and the average oocyst/egg count per gram of faeces (OPG and EPG, respectively) was calculated. Oocysts and helminth eggs detected by coprology were morphometrically identified according to Soulsby (1987) and Naciri et al. (2011).

#### Necropsy

Once faecal samples were collected from the cloaca, the proventriculus, gizzard, small and large intestines of partridges from whom the gastrointestinal tract had been collected (*n* = 547), were longitudinally opened and directly examined under a stereomicroscope for the presence of helminths. When samples included the liver (40/547), this organ was cut into slices and washed with tap water over a filter to collect any trematodes that might be present. All isolated adult helminths were preserved in 70% ethanol until identification. Adult nematodes were cleared in lactophenol and morphometrically identified according to Yamaguti (1961) and Anderson et al. (1974). Tapeworms and trematodes were stained using Semichon´s carmine (Schmidt 1986), mounted in DPX and identified according to the descriptions given by Schmidt (1986) and Yamaguti (1971).

### Statistical analysis

Prevalence (P) with 95% confidence intervals (95% CI), median abundance (MA) and median intensity (MI) with the range of detected parasites were determined according to Margolis et al. (1982) and Bush et al. (1997). Median abundance or median intensity data were calculated depending on whether all the sampled animals or only the positive ones were included in the statistical analysis, respectively. Species richness (number of parasite species in each host population) was also determined for the different climatic regions. Shapiro Wilks test was used to determine normality of parasite distributions. Fisher or Chi-square tests and the Kruskal–Wallis test were employed to compare the proportions of parasitised birds, and medians of intensity about parasite communities, respectively, according to host and environmental variables (sex, age category, bioclimatic zone). Additionally, analysis of multivariate abundance was carried out $(\mathrm{Abundance}\left[\mathrm{log}(\frac{\mathrm{y}}{\mathrm{min}}+1)\mathrm{scale}\right])$ to evaluate the distribution of the parasite species in the different bioclimatic area (Wang et al. 2012). Only nematodes and trematodes were considered, as no data on cestode abundances were calculated, since in many cases the cestode fragments found lacked scolexes, so that the exact number of cestodes could not be calculated with certainty. Significant differences were considered for *p* < 0.05. R software 3.5.2 was used to analyse the data (R Core Team 2018).

## Results

### Parasites detected by coprological analysis and necropsy

The overall prevalence of infected hosts was 47.8% (446/934). The prevalence of parasites detected by coprological analysis was 44.8% (416/927). *Eimeria* spp. oocysts and helminth eggs shedding records are summarised in Table 2. The most prevalent parasitic forms were *Eimeria* spp. oocysts, followed by nematode eggs belonging to the Ascaridida and Trichurida (*Capillaria*-like) orders.

**Table 2:** Prevalence (P), 95% confidence intervals (95%CI), median intensity (MI) and range (expressed as oocyst or eggs per gram of faeces) of parasites found by coprological analysis (*n* = 927)

	N	N + ve^(a)^	P (95%CI)	MI (range)
*Eimeria* oocysts	926	393	42.4 (39.2–45.6)	1970 (10–627,642)
Nematode eggs	927	46	4.9 (3.6–6.4)	518 (1–20,980)
Cestode eggs	570	3	0.53 (-0.07–1.12)	1 (1–1)
Trematode eggs	926	20	2.1 (1.2–3.1)	79 (1–632)

(a) N + ve: number of positive partridges

A total of 1623 helminths were collected by necropsy from 20.1% (110/547) of the red-legged partridges. Fifty-three of 547 (9.6%) partridges presented nematodes belonging to six different species and two genera, with MI (range) = 28.6 (1–239) nematodes and MA (range) = 2.7 (0–239) nematodes. Similarly, 60/547 (11%) partridges had cestodes and only 12 of the 40 individuals whose liver was available (30%) were parasitized by trematodes, with MI = 9 (1–44) and MA = 0.2 (0–44) trematodes per host. Regarding co-infections, 76.3% (84/110) of the parasitized partridges had only one helminth species, while multiparasitism with two, three and four different species was found in 19 (17.2%), 6 (5.4%) and one (0.9%) bird, respectively. The overall parasite richness found was at least 16 helminth species, considering that some parasite specimens could only be identified down to the genus level because of deterioration or because they were immature stages. All these results are summarized in Table 3.

**Table 3:** Parasite prevalence (P), 95% confidence intervals (95%CI), median intensity (MI) and range of helminths isolated from red-legged partridges by necropsy

	N + ve^(a)^	P (95%CI)	MI (range)
Nematodes (*N* = 547)			
*Ascaridia galli*	11	2 (0.83–3.19)	7 (1–28)
*Ascaridia compar*	7	1.2 (0.34- 2.22)	15 (1–46)
*Heterakis tenuicauda*	15	2.7 (1.37–4.11)	8 (1–152)
*Heterakis gallinarum*	8	1.4 (0.46–2.47)	1.5 (1–19)
*Trichostrongylus tenuis*	18	3.3 (1.79–4.78)	7.5 (1–239)
*Aonchotheca caudinflata*	2	0.36 (-1.14–1.87)	82 (1–163)
*Subulura* spp*.*	8	1.4 (0.46–2.47)	5.5 (1–55)
*Cyrnea* spp*.*	3	0.55 (-0.07–1.17)	2 (1–3)
Cestodes (*N* = 537)			
*Raillietina* spp*.*	17	3.1 (1.68–4.65)	NA
*Raillietina tetragona*	19	3.5 (1.97–5.10)	NA
*Raillietina echinobotridia*	4	0.74 (0.02–1.47)	NA
*Raillietina micracantha*	15	2.7 (1.40–4.19)	NA
*Rhabdometra nigropunctata*	3	0.56 (-0.07–1.19)	NA
*Choanotaenia infundibulum*	1	0.19 (-0.18–0.55)	NA
Trematodes (*N* = 40)			
*Dicrocoelium* spp*.*	1	2.5 (-2.34–7.34)	1 (1–1)
*Brachylaima* spp*.*	1	2.5 (-2.34–7.34)	18 (18–18)
*Brachylecithum* spp*.*	10	25 (11.58–38.42)	4 (1–44)

(a) N + ve: number of positive partridges

### Biotic and abiotic risk factors

Although sex and age data were not always available, using the existing registries, the influence of different biotic (sex and age) and abiotic (bioclimatic zone of birds’ origin) on prevalence and intensity of parasites detected by coprological analysis and necropsy are summarised in Tables 4 and 5, respectively.

**Table 4:** Influence of biotic and abiotic risk factors on prevalence and median of parasites found by coprological techniques

					*Eimeria* spp.		Nematode eggs		Cestode eggs		Trematode eggs	
Variable	Level	N^(a)^	Infected	P (95%CI) ^a^	P (95%CI)	MI (range)^(b)^	P (95%CI)	MI (range)	P (95%CI)	MI (range)	P (95%CI)	MI (range)
Sex	Female	217	106	49 (42.2–55.5)	45 (38.1–51.3)	1606 (26–627,642)	6 (2.8–9.1)	212 (1–1267)	0.46 (-0.4–1.4)	1 (1–1)	3 (0.6–4.9)	40 (1–632)
	Male	260	116	45 (38.6–50.6)	42 (35.5–47.5)	1605 (11–550,964)	5 (2.1–7.2)	564 (30–12,281)	0.38 (-0.4–1.1)	1 (1–1)	4 (1.2–5.7)	34 (1–193)
Age	Juvenile	123	61	50 (40.7–58.4)	50 (39.9–57.6)	1459 (26–550,964)	4 (0.6–7.5)	949 (38–12,281)	0.8 (-0.7–2.4)	1 (1–1)	2 (-0.6–3.9)	108 (23–193)
	Subadult	73	28	38 (27.2–49.5)	38 (25.9–48.1)	1970 (61–392,733)	3 (-1–6.5)	200 (1–400)	1.4 (-1.3–4)	1 (1–1)	8* (1.9–14.5)	37 (17–632)
	Adult	282	134	47.5 (41.7–53.3)	43 (36.4–47.9)	1218 (11–627,642)	6 (3.5–9.2)	193 (50–5537)	0.35 (-0.3–1)	1 (1–1)	3 (0.7–4.3)	40 (1–167)
Climate region^(c)^	HTA	103	37	36 (26.6–45.2)	27 (18.6–35.8)	2692 (42–442,550)	11* (4.7–16.6)	417 (20–2,020,983)	0	0	0	0
	SS	248	84	34 (27.9–39.7)	32 (26.1–37.6)	2165 (10–550,964)	3 (1–5.4)	471 (1–8942)	0	0	2 (0.3–3.8)	165 (17–188)
	SM	311	123	39.5 (34.1–44.9)	39 (33.8–44.6)	657 (25–277,333)	2 (0.4–3.4)	558 (278–1111)	0	0	1 (-0.1–2)	1 (1–1)
	T	272	173	64* (57.9–69.3)	61* (54.5–66.1)	4416 (11–627,642)*	8 (4.5–10.9)	503 (21–12,281)	1 (-0.1–2.3)	1 (1–1)	4* (2–6.8)	113 (21–632)

(a) N: Total number of partridges belonging to this variable level. Prevalence (P) and 95% confidence intervals

(b) MI (median intensity) and range (minimum and maximum values) of parasites in red-legged partridges

(c) *HTA* Humid Temperate Atlantic, *SS* Subhumid Supramediterranean, *SM* Semiarid-Mesomediterranean, *T* Thermomediterranean

(*) Significant differences between levels of the variable. Asterisk placed in level with highest value

**Table 5:** Influence of biotic and abiotic risk factors on prevalence and median of parasites found in necropsies

					Nematodes^(d)^		Cestodes		Trematodes^(e)^		Ascaridida	
Variable	Level	N^(a)^	Infected	P (95%CI)^(a)^	P (95%CI)	MI (range)^(b)^	P (95%CI)	MI (range)	P (95%CI)	MI (range)	P (95%CI)	MI (range)
Sex	Female	178	38	21 (15.3–27.4)	10 (5.2–13.9)	5 (1–46)	14* (8.9–19.1)	NA	1 (-0.4–2.7)	3 (1–5)	6 (2.6–9.7)	5 (1–46)
	Male	228	32	14 (9.5–18.5)	9 (5.1–12.4)	16 (1–59)*	7 (3.7–10.3)	NA	1 (-0.2–2.8)	18 (1–18)	5 (2.4–8.2)	22 (1–152)
Age	Juvenile	101	16	16 (8.7–22.9)	9 (3.3–14.7)	2 (1–188)	12 (5.6–18.2)	NA	0	0	7 (1.9–11.9)	1 (1–25)
	Subadult	39	5	13 (2.3–23.3)	3 (-2.4–7.5)	1 (1–1)	5 (-1.8–12)	NA	5 (-1.8–12)	11.5 (5–18)	0	0
	Adult	267	50	19 (14–23.4)	10 (6.5–13.7)	12 (1–152)	11 (6.8–14.2)	NA	1 (-0.1–2.4)	1 (1–18)	6 (3.1–8.8)	14 (1–152)
Climate region^(c)^	HTA	46	7	15 (4.8–25.6)	15 (4.8–25.6)	9 (1–29)	0	NA	0	0	13* (3.3–22.8)	14 (1–29)
	SS	86	7	8 (2.4–13.9)	3 (-0.4–7.4)	55 (1–56)	6 (0.9–10.7)	NA	0	0	2 (-0.8–5.5)	28.5 (1–56)
	SM	243	29	12 (7.8–16)	5 (1.9–7.1)	7 (1–26)	5 (1.9–7.1)	NA	4 (1.3–6)	5 (1–44)	3 (1–5.5)	7.5 (1–22)
	T	172	63	37* (29.4–43.8)	19* (12.8–24.4)	10 (1–239)	27* (19.1–32.1)	NA	2 (-0.2–3.7)	1 (1–18)	8 (4–12.2)	9.5 (1–152)

(a) N: Total number of partridges belonging to this variable level. Prevalence (P) and 95% confidence intervals

(b) Median intensity and range (minimum and maximum values) of parasites in red-legged partridges

(c) *HTA* Humid Temperate Atlantic, *SS* Subhumid Supramediterranean, *SM* Semiarid-Mesomediterranean, *T* Thermomediterranean

(d) Except Ascaridida

(e) These results were obtained from 40 individuals

(*) Significant differences between levels of the variable

Sub-adult partridges showed significantly higher prevalence of trematode eggs than the other age groups. Regarding helminth detection by necropsy, males presented higher intensity of nematodes than females, whereas cestode prevalence was, by contrast, significantly higher in females. Finally, regarding the bioclimatic zones, the prevalence of partridges with *Eimeria* spp. oocyst and median intensity of oocyst, as well as the prevalence of trematode eggs, cestodes and nematodes (except Ascaridida order), were significantly higher in the Thermomediterranean area. On the contrary, the prevalence of Ascaridida helminths found by necropsy, as well as Trichurida and Ascaridida eggs detected by coprological techniques, were significantly higher in the Humid Temperate Atlantic area (Tables 4 and 5), as is the case of *A. galli* abundances. Specifically, 46 birds were collected from this bioclimatic zone, and six of them had these nematodes, so prevalence is higher compared to that found in the Semiarid-Mesomediterranean area (8/243) or in the Thermomediterranean area (14/172). On the other side, regarding Thermomediterranean area, only *T. tenuis* abundances were statistically significant (*p* < 0.05).

**Fig. 2:**
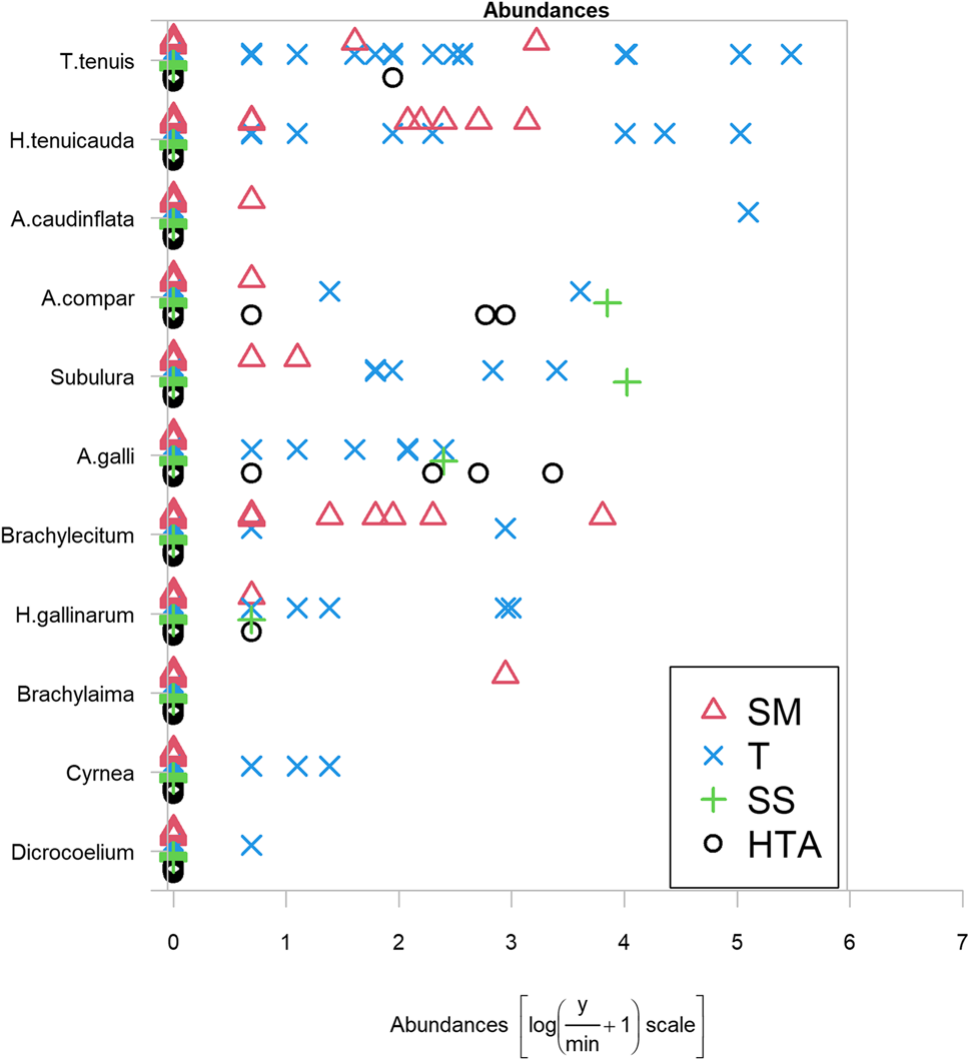
Abundances of nematode and trematode species found in red-legged partridges according to the climatic region of origin

In general terms, the mean abundance of nematodes and trematodes was statistically significant higher in southern areas (Semiarid-Mesomediterranean and Thermomediterranean areas) (*p* < 0.05) (Fig. 2). Likewise, the parasite richness was greater in the Semiarid-Mesomediterranean (13 species) and Thermomediterranean (15 species) regions, where the richness of nematodes represents around half of the species. The parasitic species found in all the areas, was *Eimeria* spp. as shown in Table 6.

**Table 6:** Red-legged partridge parasite richness according to climate region provenance

		Presence/absence^(a)^ of species in each climate region^(b)^			
	Parasite species	HTA	SS	SM	T
	*Eimeria* spp.	+	+	+	+
Nematodes	*A. galli*	+	+	-	+
	*A. compar*	+	+	+	+
	*H. gallinarum*	+	+	+	+
	*H. tenuicauda*	-	-	+	+
	*T. tenuis*	+	-	+	+
	*A. caudinflata*	-	-	+	+
	*Cyrnea* spp.	-	-	-	+
	*Subulura* spp.	-	+	+	+
	Richness	**4**	**4**	**6**	**8**
Cestodes	*R. tetragona*	-	+	+	+
	*R. echinobotridia*	-	+	+	+
	*R. micracantha*	-	+	+	+
	*R. nigropunctata*	-	+	+	-
	*C. infundibulum*	-	-	-	+
	Richness	0	4	4	4
Trematodes	*Dicrocoelium* spp.	-	-	-	+
	*Brachylaima* spp.	-	-	+	-
	*Brachylecithum* spp.	-	-	+	+
	Richness	0	0	2	2
	Total species	**5**	**9**	**13**	**15**

(a) Presence ( +) and absence (-)

(b) *HTA* Humid Temperate Atlantic, *SS* Subhumid Supramediterranean, *SM* Semiarid-Mesomediterranean, *T* Thermomediterranean

## Discussion

The results of this epidemiological investigation show a rich parasite community in Spanish red-legged populations, with most of the helminth species found having been previously described in this host. Considering the detection of parasites by coprological analysis and/or necropsy, the overall parasite prevalence found was 47.8%.

Focusing on the results obtained by coprological analysis, the overall prevalence was 44.8% (416/927), similar to the 38% previously described by Millán et al. (2003) in captive and wild red-legged partridges. Regarding the prevalence of helminths found by necropsy, it was 20.1% (110/547), a low value when compared to the prevalence of 69% (161/235) reported by Calvete et al. (2004) in red-legged partridges from two provinces of central Spain (Toledo and Ciudad Real), both with an important tradition of game bird hunting. These differences may be due to factors as the density of birds at the sampled site, land use characteristics as suggested by Calvete et al. (2004), or climatic features that influence on the survival of infective parasite stages and/or the distribution of their intermediate and paratenic hosts.

The prevalence of trematode infections was low when compared to the studies previously mentioned (Calvete et al. 2004). In our study, the most prevalent trematode detected by necropsy was *Brachylecithum* spp., a liver fluke that has been previously detected in red-legged partridges (Millán 2009). *Dicrocoelium* spp. was detected in only 2.5% of necropsied livers, while it was the most prevalent genus in hunted wild red-legged partridges studied by Millán et al. (2004a) and Calvete et al. (2003), who described prevalences of 46.8% and 17.3%, respectively. The low *Dicrocoelium* spp. prevalence that we found is probably due to the small number of livers available for our study. In addition, although we detected eggs of trematodes by coprological analysis, we are probably underestimating the prevalence of flukes, since the sensitivity of coprological techniques is lower than the detection of trematode specimens by necropsy, especially in the case of liver flukes (Sithithaworn et al. 1991; Sobral et al. 2019). The prevalence of *Brachylaima* spp. was 2.5%, similar to the findings described by Millán et al. (2004a), who considered these digestive flukes as anecdotal.

Among the helminths detected by necropsy, the prevalence of cestodes was the highest, especially in the case of the genus *Railletina* spp. (3.1%) and, in particular, to *R. tetragona* and *R. micrachanta* (3.5% and 2.7%, respectively). *R. tetragona* was also the most prevalent cestode in the study carried out by Calvete et al. (2003) and Millán et al. (2004a). *Rhabdometra nigropunctata* (4/538) and *Choanotaenia infundibulum* (1/538) prevalence was lower than 1%. These tapeworm species, as well as *R. tetragona*, were detected in wild red-legged partridges from the Iberian Peninsula in previous studies (Calvete et al. 2003; Millán et al. 2004a). Although the prevalence of adult tapeworms by necropsy was 11%, the prevalence of cestodes in partridges was much lower (1%) by coprological analysis, indicating a low diagnostic sensitivity of this last technique, as has been demonstrated by other authors (Zloch et al. 2021).

These results on trematode and cestode prevalence suggest that the parasitized partridges were either wild individuals or, alternatively, farm-reared birds released in the months prior to hunting that, during that time, acquired the infection in the wild (Villanúa et al. 2008). In this regard, it is noteworthy that 25–34% of the released red-legged partridges died during the first 72 h post-release and that, in general, survival rate is very low due to predation, hunting and starvation (Gortázar et al. 2000; Pérez et al. 2004).

Ascaridida was the most prevalent nematode group (7.5% of animals), including *Heterakis* spp. (4.2%) and *Ascaridia* spp. (3.3%), nematodes that have been described with low prevalences in red-legged partridges from Spain and Italy (Millán 2009; Polello et al. 2021).

*Trichostrongylus tenuis* was recovered from 3.3% of the red-legged partridges necropsied, while other studies have reported prevalences of 12–13% (Calvete et al. 2004; Millán et al. 2004a). It is a parasite that, despite having a direct life cycle, is considered more prevalent in wild than in farm-reared partridges, because it is unable to complete its life cycle when there is little vegetation in the environment, as happens in breeding farms (Millán et al. 2004a). In case of indirect life cycle nematodes as *Aonchotheca caudinflata*, *Cyrnea* spp. and *Subulura* spp., the prevalence detected was less than 1%. In other studies, similar values of prevalence from wild specimens have been reported: 1.8% (Calvete et al. 2003, 2004; Millan et al. 2004a). Hence, as noted above, these parasites could have been acquired by wild individuals or partridges that had lived in the wild during the first periods of life.

Ecological determinants and anthropogenic factors, such as the type of management implemented, influence the prevalence, intensity and parasite richness in game species (Tompkins et al. 1999; Poulin 2007; González-Quevedo et al. 2014; Morand 2015; Fanelli et al. 2020). In our study, we have included red-legged partridges from 15 provinces, ranging from the north to the south of the Iberian Peninsula, so it can be assumed that the greater heterogeneity of the bioclimatic regions sampled, each with its own characteristics of land use and management of the species, has influenced our results.

*Eimeria* spp. was the most prevalent parasite in this study, as in previous surveys with other Galliformes species (Santilli and Bagliacca 2012; Globokar et al. 2017; Polello et al. 2021). *Eimeria* spp. and Ascaridida parasites are frequently found in farm-reared partridge populations (Bolognesi et al. 2006; Villanúa et al. 2008; Millán 2009; Naciri et al. 2011; Máca and Pavlásek 2020). In other game birds, such as pheasants, individuals from restocking areas have been found to have twice the prevalence of *Eimeria* spp. as those from non-repopulated areas, 51.3% and 25.6%, respectively (Santilli and Bagliacca 2012). In agreement, Mani et al. (2000) pointed out that wild pheasant had lower parasite prevalence than those hunted in areas where farm-reared birds are frequently released. In fact, coccidiosis is one of the main problems in partridge farms, where the characteristics of the facilities provide conditions that help to spread infection (Naciri et al. 2011). On the other hand, Albendazole is usually administered in red-legged partridge farms to limit the infection with nematodes, mainly Trichurida and Ascaridida, although results reported by Villanúa et al. (2008) have demonstrated the limited efficacy of this drug, in particular with regard to *Heterakis* spp. This scenario coincides with our results since *Eimeria* spp. was the most prevalent genus detected with coprological techniques, whereas *Ascaridia* spp. and *Heterakis* spp. were the most prevalent helminths detected by necropsy. Moreover, according to the bioclimatic zone of partridge’s origin, our results indicate that those birds coming from the Thermomediterranean area showed higher prevalence of *Eimeria* spp. (60.3%; 164/272) and higher number of oocysts shedding (considered as a proxy of median parasite intensity) than partridges from other bioclimatic zones. These results could be the consequence of more frequent restocking with farm-reared birds in hunting estates from the Thermomediterranean area. Hunting is an important tradition in Spain, being especially significant in centre and south areas (Viñuela et al. 2013). In fact, game crops are used as a tool to provide supplementary food, nesting cover or protection for game birds in Mediterranean areas, characterized by dry and hot summers (Reino et al. 2016). Due to the climatic characteristics of the Iberian Peninsula, annual periods of drought are common and, in order to palliate this, the set-up of water points in hunting areas is also a frequently used management measure (Gaudioso Lacasa et al. 2010). These types of practices, which has been widely applied for decades in the game management of partridges, leads to the establishment of hot spots for pathogen transmission, mostly in water stress periods. In this sense, Gaudioso Lacasa et al. (2010) observed that water points were used by both wild and game birds, especially in summer, when chicks need more requirements. As with supplementary feeding, these management measures promote parasite transmission and disease outbreaks due to higher contact among birds (Villanúa et al. 2006; Millán 2009). Therefore, both the frequent release of farm-reared partridges in Southern areas of Spain to cover the needs of the hunters, and the management practices used in these hunting estates, could determine the high *Eimeria* spp. prevalence and the highest presence of Ascaridida among all the helminths found in partridges coming from these areas.

Regarding biotic factors, cestode prevalence was significantly higher in female partridges, whilst the median intensity of nematodes was greater in males. On the other hand, sub-adult partridges showed a significantly higher prevalence of trematode eggs in faeces. Other studies have shown differences in parasite intensity and prevalence depending on the sex and the age of the host in a broad range of species (Poulin 2007; Martínez-Guijosa et al. 2015). Several reasons could account for gender-related differences in parasite infections. For instance, testosterone production in males is energy demanding and has been linked to immunosupression and increased probability of becoming parasitized (Klein 2004; Guerra-Silveira and Abad-Franch 2013). Other studies attributed differences in the risk of parasite infection between males and females to food preferences (Provencher et al. 2016). Immunosuppression and food preferences have also been associated to age-related differences in the prevalence of parasite infections (Thieltges et al. 2006).

Finally, considering parasite richness, a total of 16 species of helminths were described by necropsy in this study. These included eight species of nematodes, five of cestodes and three of trematodes (Table 3). The Thermomediterranean area was the one with highest richness of parasite species (15), followed by the Semiarid-Mesomediterranean (14), the Supramediterranean (8), and finally the Humid Temperate Atlantic area (4), which had the lower richness (Table 6). Brown (1984) suggested that abundance and distribution of helminths reflect the abundance and distribution of their hosts, and Calvete et al. (2003) propose that the distribution of parasites, in general, is related to the distribution of their intermediate and definitive hosts. In this sense, socio-economic interest around game birds in the southern Iberian Peninsula, where release of farm-reared partridges as well as management practices (use of game crops or water points) are more frequent than in other territories, could explain the results obtained about *Eimeria* spp. or Ascaridida infections in the Thermomediterranean area, as well as the higher values of the abundance of *T. tenuis* comparing with the other bioclimatic areas.

Moreover, the environmental characteristics of each area may also cause the parasite richness between areas to differ. In fact, results about multivariate abundance indicate statistically significant differences among bioclimatic zones, with Semiarid-Mesomediterranean and Thermomediterranean areas showing the highest rates of trematodes and nematodes, respectively. In this sense, the presence of indirect life cycle parasites and the high rate of parasite richness reached in these areas, could be due to the influence of environmental factors in this bioclimatic zone on parasites using intermediate and paratenic hosts in their life cycles (Krasnov and Poulin 2010; Morand 2015).

Climate has been related to the environmental persistence of parasite free stages, and also determines the abundance and distribution of intermediate and paratenic hosts (Holand et al. 2019). Coleoptera, Diptera, Hymenoptera and other invertebrate parasite hosts constitute an important part of the partridge`s diet (Holland et al. 2006); in particular, dung beetles play an important role in parasite transmission (Nichols and Gómez 2014). Those belonging to the Aphodiia, Scarabaeidae or Geotrupidae families are present in Iberian Peninsula, and the latter two families comprise dung species that are well adapted to arid environments (Verdú and Galante 2002; Cabrero-Sañudo and Lobo 2003). Additionally, dry lands or calcareous soils typical in Mediterranean ecosystems are favourable for terrestrial snails or ants acting as intermediate host of trematodes (Manga-González et al. 2001; Otranto and Traversa 2003). In fact, fluke species are only present in Semiarid-Mesomediterranean and Thermomediterranean areas. In addition, the environmental characteristics of the Humid Temperate Atlantic area provide adequate requirements for earthworms’ development (Edwards and Lofty 1977), which are intermediate or paratenic hosts for Trichurida and Ascaridida nematodes, respectively (Fedynich 2008; Yabsley 2008). Therefore, the presence of earthworms could be helping the maintenance of these parasites in Humid Temperate Atlantic areas.

## Conclusions

Our study provides an account of the prevalence, richness, abundance and intensity of parasite infections in red-legged partridges in the main bioclimatic territories in the Iberian Peninsula. This novel information about the geographic distribution of digestive parasites in this game species is necessary to evaluate, in a comprehensive manner, the health status of the Iberian populations. In this way, a deeper knowledge of the distribution and prevalence of these parasites (some of which potentially cause of a negative impact on partridges), is the necessary basis to design management practices that limit the spread of these pathogens and, consequently, to ensure the long-term sustainability of red-legged partridge. Special emphasis should be placed on controlling parasite infections in farm-reared partridges prior to restocking hunting estates, as well as avoiding parasite concentration in areas where supplementary feeding is provided following their introduction.

## Data Availability

The datasets generated during and/or analysed during the current study are available from the corresponding author on reasonable request.
